# Supplementary material for: Microsatellite Analysis Revealed Potential DNA Markers for Gestation Length and Sub-Population Diversity in Kari Sheep
Source: Animals (Basel). 2022 Nov 25;12(23):3292. doi: 10.3390/ani12233292 (PMC9736151; doi:10.3390/ani12233292)
Supplement: Supplementary file 1 [file animals-12-03292-s001.zip › animals-1993430-supplementary.pdf]

Table S1: Primer sequences for microsatellite DNA markers recommended for genetic diversity studies in sheep.

Name	Primer sequence forward and reverse	Annealing temp	Chromosome location	Map distance cM
MAF65	AAAGGCCAGAGTATGCAATTAGGAG<br>CCACTCCTCCTGAGAATATAACATG	60°C	15	31.2
OarFCB193	TTCATCTCAGACTGGGATTCAGAAAGGC<br>GCTTGGAAATAACCCTCCTGCATCCC	54°C	11	67.8
OarJMP29	GTATACACGTGGACACCGCTTTGTAC<br>GAAGTGGCAAGATTCAGAGGGGAAG	56°C	24	4.5
OarJMP58	GAAGTCATTGAGGGGTCGCTAACC<br>CTTCATGTTCACAGGGTCAGGG	58°C	26	50.3
OarFCB304	CCCTAGGAGCTTTCAATAAAGAATCGG<br>CGCTGCTGTCAACTGGGTCAGGG	56°C	19	66.3
BM8125	CTCTATCTGTGGAAAAGGTGGG<br>GGGGGTTAGACTTCAACATACG	50°C	17	80.2
OarFCB128	ATAAAGCATCTTCTCTTTATTTCCTCGC<br>CAGCTGAGCAACTAAGACATACATGCG	55°C	2p	96.0
OarCP34	GCTGAACAATGTGATATGTTCAGG<br>GGGACAATACTGTCTTAGATGCTGC	50°C	3p	39.1
OarVH72	GGCCTCTCAAGGGGCAAGAGCAGG<br>CTCTAGAGGATCTGGAATGCAAAGCTC	57°C	25	44.5
OarHH47	TTTATTGACAAACTCTCTTCCTAACTCCACC<br>GTAGTTATTTAAAAAAATATCATACCTCTTAAG	58°C	18	77.7
DYMS1	AACAACATCAAACAGTAAGAG<br>CATAGTAACAGATCTTCCTACA	59°C	20	11.6
SRCRSP1	TGCAAGAAGTTTTTCCAGAGC<br>ACCCTGGTTTCACAAAAGG	54°C	Unknown	
SRCRSP5	GGACTCTACCAACTGAGCTACAAG<br>GTTTCTTTGAAATGAAGCTAAAGCAATGC	56°C	21	
SRCRSP9	AGAGGATCTGGAAATGGAATC<br>GCACTCTTTTCAGCCCTAATG	55°C	12	
MCM140	GTTCGTACTTCTGGGTACTGGTCTC<br>GTCCATGGATTTGCAGAGTCAG	60°C	6	93.4
MAF33	GATCTTTGTTTCAATCTATTCCAATTTC<br>GATCATCTGAGTGTGAGTATATACAG	60°C	9	60.0
MAF209	GATCACAAAAAGTTGGATACAACCGTGG<br>TCATGCACTTAAGTATGTAGGATGCTG	63°C	17	46.7
INRA63	ATTTGCACAAGCTAAATCTAACC<br>AAACCACAGAAATGCTTGGAAG	58°C	14	62.9
OarFCB20	AAATGTGTTTAAGATTCCATACAGTG<br>GGAAAACCCCCATATATACCTATAC	56°C	2q	190.2
BM1329	TTGTTTAGGCAAGTCCAAAGTC<br>AACACCGCAGCTTCATCC	50°C	6	42.2
MAF214	GGGTGATCTTAGGGAGGTTTTGGAGG<br>AATGCAGGAGATCTGAGGCAGGGACG	58°C	16	43.9

ILSTS11	GCTTGCTACATGGAAAGTGC<br>CTAAAAGTCAGAGCCCTACC	55°C	9	41.0
MCM527	GTCCATTGCCTCAAATCAATTC<br>AAACCACTTGACTACTCCCCAA	58°C	5	120.3
OarFCB226	CTATATGTTGCCTTTCCCTTCCTGC<br>GTGAGTCCCATAGAGCATAAGCTC	60°C	2	44.9
ILSTS28	TCCAGATTTTGTACCAGACC<br>GTCATGTCATACCTTTGAGC	53°C	3	34.0
MAF70	CACGGAGTCACAAAGAGTCAGACC<br>GCAGGACTCTACGGGGCCTTTGC	60°C	4	51.6
BM1824	GAGCAAGGTGTTTTTCCAATC<br>CATTCTCCAACTGCTTCCTTG	58°C	1q	292.5
OarAE129	AATCCAGTGTGTGAAAGACTAATCCAG<br>GTAGATCAAGATATAGAATATTTTTCAACACC	54°C	5	115.4
HUJ616	TTCAAATACACATTGACAGGG<br>GGACCTTTGGCAATGGAAGG	54°C	13	2.5
OarCP38	CAATTTGGTGCATATTCAAGGTTGC<br>GCAGTCGCAGCAGGCTGAAGAGG	52°C	10	93.3
ILSTS5	GGAAGCAATGAAATCTATAGCC<br>TGTTCTGTGAGTTTGTAAGC	55°C	7	127.1

Table S2. Shared and unique alleles in Kari subtypes.

Locus	Number of alleles	Shared alleles				Unique alleles		
		**S-M**	**S-L**	**M-L**	**S-M-L**	**S**	**M**	**L**
OarJMP58	12	4	3	7	3	2		2
INRA63	12	6	4	8	4			
OarVH72	11	5	7	5	4		1	1
DYMS1	10	6	6	7	6	1		2
OarFCB304	8	2	1	4	1	2	1	
ILSTS11	8	2	0	1	0	1	1	2
OarJMP29	8	5	5	5	4		1	
SRCRSP1	8	2	1	4	1		1	2
MAF214	7	4	3	3	2			1
BM1824	7	5	4	6	4			
MCM527	7	2	4	4	2			1
MCM140	7	4	3	5	3	1	1	
ILSTS5	7	4	2	4	2			1
BM1329	7	2	3	4	2			1
SRCRSP5	7	3	2	4	2	1		
HUJ616	6	2	2	4	2		1	1
OarFCB226	6	2	2	5	2	1		
MAF33	5	2	3	2	3	1		1
SRCRSP9	5	3	4	4	3			
BM8125	4	3	1	1	1		1	
MAF65	4	4	2	2	2			
OarFCB193	2	1	1	2	1			
**Total**	**158**	**73**	**63**	**91**	**54**	**10**	**8**	**15**

Key: S = Kari-S; M = Kari-M; L = Kari-L.

Table S3: Allele sizes and their frequencies in different Kari sheep subtypes

Locus	Allele	Size	Population			Locus	Allele	Size	Population		
			Kari-S	Kari-M	Kari-L				Kari-S	Kari-M	Kari-L
OarJMP58	A	123			**0.083**	INRA63	A	155		0.046	0.136
	B	126			**0.042**		B	158	0.083	0.273	
	C	129		0.050	0.083		C	166	0.167	0.046	0.227
	D	132	**0.167**				D	170		0.182	0.046
	E	135		0.050	0.125		E	174	0.083	0.091	0.091
	F	138		0.200	0.125		F	178		0.046	0.091
	G	141	0.250	0.050	0.167		G	182	0.167	0.046	
	H	144		0.200	0.083		H	190	0.167	0.046	0.136
	I	148	0.167	0.100	0.125		I	195		0.182	0.091
	J	158	0.250	0.250	0.167		J	199	0.167	0.046	0.046
	K	178	0.083	0.100			K	209	0.167		0.136
	L	198	**0.083**				L	219			
	Null						Null				
OarVH72	A	126			**0.083**	DYMS1	A	158	**0.083**		
	B	129	0.083		0.083		B	162		0.091	0.227
	C	132	0.167	0.375	0.083		C	170			**0.046**
	D	135	0.167	0.062	0.041		D	178	0.083	0.136	0.091
	E	138		0.125	0.125		E	182	0.333	0.136	0.182
	F	144	0.083		0.167		F	190			**0.136**
	G	148	0.167	0.062			G	195	0.083	0.318	0.091
	H	151	0.167	0.062	0.167		H	204	0.167	0.045	0.091
	I	155	0.083	0.250	0.083		I	209	0.167	0.045	0.046
	J	158		**0.062**			J	219	0.083	0.227	0.091
	K	162	0.083		0.167		Null				0.017
	Null				0.070						
OarFCB304	A	144	**0.125**			ILSTS11	A	275	0.250	0.182	
	B	155	**0.250**				B	282	**0.250**		
	C	158	0.250	0.312	0.200		C	295		0.455	0.357
	D	162		0.062	0.300		D	302		**0.182**	
	E	166		**0.187**			E	316			**0.287**
	F	170	0.375	0.062			F	331	0.500	0.136	
	G	178		0.250	0.100		G	331		0.045	0.074
	H	182		0.125	0.400		H	339			**0.287**
	Null			0.100	0.205		Null			0.200	0.249
OarJMP29	A	127	0.100	0.222	0.083	SRCRSP1	A	112			**0.083**
	B	132	0.100	0.111	0.250		B	117			**0.208**
	C	145		0.333	0.167		C	123		0.350	0.208
	D	150	0.400	0.056			D	129		0.150	0.167
	E	158	0.100	0.111	0.167		E	132	0.500	0.250	0.167
	F	165	0.200	0.111	0.083		F	141		0.050	0.167
	G	172	0.100		0.250		G	148		**0.100**	
	H	180		**0.056**			H	151	0.500	0.100	
	Null			0.093			Null			0.123	0.043

Locus	Allele	Size	Population			Locus	Allele	Size	Population		
			Kari-S	Kari-M	Kari-L				Kari-S	Kari-M	Kari-L
MAF214	A	339	0.333	0.636	0.208	BM1824	A	170	0.167	0.091	
	B	347	0.417	0.045			B	174		0.546	0.056
	C	407			**0.292**		C	178	0.583	0.046	0.722
	D	426	0.083	0.182	0.042		D	186	0.083	0.091	0.056
	E	436		0.091	0.375		E	190	0.083	0.136	0.056
	F	447	0.083	0.045			F	199		0.046	0.056
	G	457	0.083		0.083		G	229	0.083	0.046	0.056
	Null		0.152		0.105		Null		0.137	0.066	0.163
MCM527	A	158			**0.136**	MCM140	A	158		0.250	0.071
	B	162		0.167	0.227		B	174		**0.150**	
	C	166	0.167		0.045		C	178	0.167	0.100	0.071
	D	170	0.333	0.333	0.136		D	182	0.333	0.250	0.357
	E	186	0.167		0.273		E	190	0.167	0.200	0.143
	F	190		0.167	0.045		F	200		0.050	0.357
	G	195	0.333	0.333	0.136		G	209	**0.333**		
	Null				0.071		Null		0.232	0.051	
ILSTS5	A	186		0.250	0.090	BM1329	A	209	0.333	0.400	0.250
	B	195	0.250	0.150			B	214		0.100	0.125
	C	200	0.250	0.100			C	219	0.167		0.062
	D	214		0.250	0.273		D	245		0.400	0.125
	E	219	0.167	0.200	0.045		E	257	0.333	0.100	0.187
	F	224	0.333	0.050	0.318		F	269	0.167		0.062
	G	240			**0.201**		G	288			**0.188**
	Null						Null				0.214
SRCRSP5	A	148	0.200	0.100	0.167	HUJ616	A	117			**0.250**
	B	151	0.300	0.550	0.542		B	120	0.500	0.300	0.125
	C	158		0.050	0.125		C	126		0.100	0.125
	D	174	**0.200**				D	132		0.100	0.125
	E	178	0.300	0.100			E	138	0.500	0.300	0.375
	F	182		0.100	0.125		F	148		**0.200**	
	G	186		0.100	0.042		Null			0.133	
	Null			0.161	0.252						
OarFCB226	A	110		0.143	0.143	MAF33	A	120	0.167	0.500	0.350
	B	117	0.833	0.143	0.357		B	132	0.333	0.500	0.300
	C	120		0.149	0.214		C	138			**0.250**
	D	129		0.143	0.214		D	144	0.167		0.100
	E	151	0.083	0.143	0.071		E	155	**0.333**		
	F	174	**0.083**				Null				
	Null		0.251	0.095	0.126						
SRCRSP9	A	110		0.350	0.318	BM8125	A	110	0.375	0.333	1.000
	B	112	0.667	0.350	0.272		B	112	0.125	0.167	
	C	117	0.083	0.100	0.136		C	117		**0.333**	
	D	126	0.167	0.200	0.181		D	123	0.500	0.167	
	E	135	0.083		0.091		Null		0.558	0.217	
	Null		0.143	0.033	0.273						

Locus	Allele	Size	Population			Locus	Allele	Size	Population		
			Kari-S	Kari-M	Kari-L				Kari-S	Kari-M	Kari-L
MAF65	A	79	0.500	0.636	0.667	OarFCB1 93	A	79		0.364	0.091
	B	110	0.300	0.273	0.333		B	100	1.000	0.636	0.909
	C	132	0.100	0.046			Null			0.798	0.953
	D	144	0.100	0.046							
	Null		0.072	0.061							

Bold numbers indicate unique alleles
